# Contrast‑induced encephalopathy: An allergological point of view

**DOI:** 10.1016/j.radcr.2025.10.007

**Published:** 2025-11-17

**Authors:** Patricia Andrade Garbán, Marta Goyanes Malumbres, Carolina Calvo Corbella, María González Labrador, Raquel Izquierdo-Fernández, Diego Mayo-Canalejo, Sabela Pérez-Codesido, Miguel Ángel Tejedor-Alonso

**Affiliations:** a Allergy Unit, Hospital Universitario Fundación Alcorcón, Calle Budapest 1, 28922, Alcorcón, Spain; b Radiology Unit, Hospital Universitario de Móstoles, Móstoles, Spain; c Neurology Unit, Hospital Universitario de Móstoles, Móstoles, Spain; d Division of Immunology and Allergology, University Hospitals and Medical Faculty of Geneva, Geneva, Switzerland; e Department of Medical Specialties and Public Health, Universidad Rey Juan Carlos, Alcorcón, Spain

**Keywords:** Hypersensitivity reaction, Contrast‑induced encephalopathy, Iodinated contrasts, Atypical reactions, Allergological study

## Abstract

The intravascular administration of iodinated contrast agents (IC) can be associated with rare neurological complications such as contrast-induced encephalopathy (CIE). CIE has been primarily reported following cardiac and cerebrovascular angiographic procedures and after endovascular treatment of aneurysms. A 61-year-old woman with a history of breast cancer and recurrent respiratory infections presented with respiratory symptoms and persistent fever despite outpatient antibiotic therapy. A thoracic AngioCT with Iomeprol ruled out pulmonary thromboembolism, suggesting an infection. A few hours later, the patient developed acute confusion, behavioral changes, incoherent speech, and an erythematous rash on the trunk and proximal limbs. She was admitted to exclude ischemic, hemorrhagic, and infectious causes. A non-contrast cranial CT and lumbar puncture showed no significant findings. Other tests, including blood work, venous blood gas, and SARS-CoV-2 PCR, were normal. The patient improved spontaneously within 48 hours. A subsequent cerebral MRI with gadolinium showed no abnormalities. Given the likely association with contrast administration, the patient was referred to our Allergy Clinic. An allergological evaluation was performed, including patch and skin prick tests with Iohexol, Iodixanol, Iobitridol, Iomeprol, and Ioversol. All tests returned negative results at the 48- and 96-hour readings. The patient was discharged with a recommendation to avoid IC, except in very exceptional circumstances. CIE is a very rare, acute, and generally transient complication following the administration of IC. We highlight the importance of being aware of this adverse effect of IC, as it can lead to consultations with allergists.

## Case report

A 61-year-old woman with a history of breast cancer and multiple hospitalizations for pneumonia and bronchiectasis infections was evaluated in the emergency room due to persistent fever. Various diagnostic tests were performed, including a thoracic angioCT scan prompted by an elevated D-dimer level. During the scan, 50 milliliters of intravenous ionated contrast agents (IC) (Iomeprol) were administered at a rate of 5 mL/sec, which ruled out pulmonary thromboembolism but revealed a ground-glass image suggestive of superinfection.

Within hours of the thoracic angioCT scan, the patient showed sudden confusion, behavioral changes, and incoherent speech, along with the appearance of a maculopapular exanthem (MPE) on the trunk and extremities, despite having a normal neurological evaluation and stable vital signs.

**Fig. 1:**
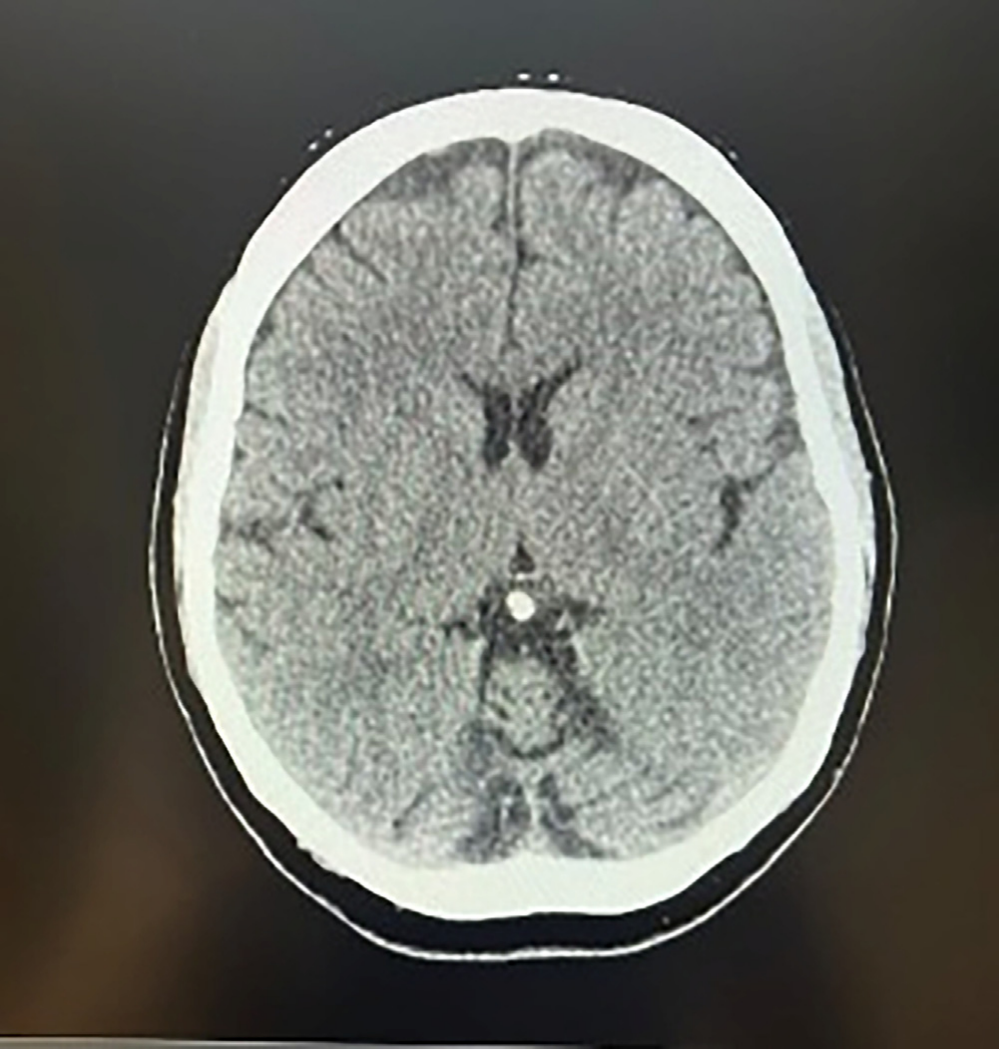
Non-contrast cranial CT scan, conducted after the onset of neurological symptoms, showed no signs of intracranial pathology.

The patient was admitted to the Neurology Department for further assessment. Acute stroke, intracranial hemorrhage, and central nervous system infection were excluded following a non-contrast cranial CT scan (Fig. 1) and a lumbar puncture, both of which showed no significant abnormalities. The results of the other tests performed are listed in Table 1.

**Table 1:** Additional tests performed during the neurology department admission.

Test	Results
Urine analysis	pH 5.5, Proteins 15 mg/dL, Glucose: negative, Nitrates: negative, red blood cells: negative.
Blood test	Normal blood count, Glucose 101 mg/dL, Creatinine 0.8 mg/dL, EPI 82 mL/min/1,73 m^2^, Sodium 134 mEq/L, Potassium 4.4 mEq/L, INR 1.01, APPT 28, D-dimer 2290, PCR 7.3 mg/L, Procalcitonin 0.22mg/mL. Normal CPK, Bilirubin, Troponins, GOT, and GPT.
Venous blood gas analysis	pH 7.35, pCo2 51 mmHg, HCO3 28.3 mmol/L, Eb 2.7mmol/L, Normal Lactate.
PCR SARS-CoV2 test	Negative.
CSF	- Red blood cells 30/uL, Leukocytes 1/uL, Glucose 60 mg/dL, Proteins 146.8 mg/dL. - Pre- and post-centrifugation liquid: Clear-transparent. - Negative culture.
PCR for Viruses and Bacteria in CSF	- Negative for *S. pneumoniae, N. meningitidis, S. agalactiae, L. monocytogenes, C. neoformans, E. coli K1, H. influenzae.* - Not detected: *Parechovirus, Cytomegalovirus, Enterovirus, Herpes simplex virus type 1 or type 2, Human herpes virus 6, Varicella Zoster Virus.*

Abbreviations: CSF, cerebral spinal fluid.

Throughout her hospitalization, the patient showed positive progress and did not require any treatment other than the antibiotics started in the emergency department for the respiratory infection. Complete and spontaneous resolution of symptoms occurred within 48 hours, with no reemergence of skin lesions, confusion, or other neurological symptoms. Before discharge, a brain MRI was performed using paramagnetic contrast (Gadoteridol), which was well-tolerated and revealed no significant abnormalities except for minor developmental venous anomalies unrelated to the episode.

Considering the timeline and the probable correlation of symptoms with the IC administration, as well as the subsequent exclusion of other neurological causes, the patient was referred to the allergology department for assessment. The patient reported no prior reactions associated with the use of ICs. An allergological examination was conducted with patch tests and prick tests involving different IC agents: Iohexol, Iodixanol, Iobitridol, Iomeprol, and Ioversol, all yielding negative results after readings at 48 and 96 hours. Since the patient refused to undergo intradermal testing, the allergy evaluation was incomplete. In our case, we did not propose a controlled challenge test.

At discharge, it was recommended to prioritize the use of diagnostic tests with paramagnetic contrasts over IC. In cases where the risk of not performing the study with IC exceeds the risk of a new CIE episode, it is recommended to use an iso-osmolar iodinated contrast at the minimum effective dose, infused at a rate of less than 5ml/sec, along with intravenous hydration 12 hours before and after IC administration. The patient has not required any further diagnostic tests or therapeutic procedures involving IC.

## Discussion

The intravascular administration of IC can be associated with rare neurological complications such as contrast-induced encephalopathy (CIE) [[1], [2], [3], [4], [5], [6]]. CIE has been mainly reported following diagnostic angiographic procedures and endovascular treatment of aneurysms [1,2,7].

CIE is a rare, acute, and typically transient complication that can occur after intravascular administration of IC [[1], [2], [3], [4]]. Its occurrence is infrequent [3,5,8], and potential risk factors include male gender, advanced age, various comorbidities, and a personal history of prior reactions to ICs [[2], [3], [4], [5], [6], [7], [8], [9]].

It is known that all IC (ionic, non-ionic, hypo-osmolar, iso-osmolar, and hyper-osmolar) can cause CIE [1,7,9]. However, a correlation appears to exist between the increased osmolality of the IC compared to blood osmolarity and the volume administered, which contributes to a higher risk of developing this condition [3,7,9].

The pathophysiology is not fully understood; however, among the proposed mechanisms, the most accepted is a possible disruption of the blood-brain barrier that allows the IC to penetrate the central nervous system, resulting in direct neuronal toxicity [[1], [2], [3], [4], [5], [6],^8^,^9^].

Clinical manifestations include symptoms of encephalitis, focal neurological deficits, seizures, transient global amnesia, and transient cortical blindness [[1], [2], [3], [4], [5], [6],^8^,^9^], with the latter being the most frequently reported [1,3,4,6]. Symptoms typically appear shortly (minutes to hours) after IC administration and completely resolve within 48-72 hours spontaneously [1,2,4,8], although more prolonged and fatal cases have been reported [3,4,8]. The most frequent radiological findings include transient cerebral edema and cortical enhancement; however, cases with normal imaging studies, like ours, have also been reported [7].

Because of the variability in its clinical presentation, a differential diagnosis with more common neurological conditions should be considered. Consequently, the diagnosis should take into account the timing of symptom onset after IC administration (minutes to hours), the transient nature of the condition (which usually resolves completely within 48-72 hours), and the need to rule out other neurological, pharmacological, or metabolic causes [1,4].

Similar to our patient, in the few published clinical cases, treatment remains symptomatic [[5], [6], [7],^9^], although some have received intravenous corticosteroids, whose use is debated [5].

To date, recommendations for these patients remain unclear, as there are reports of recurrence upon subsequent exposures [1,4,7]. In contrast, others have demonstrated good tolerance to re-exposure to the implicated IC [4,9]. Nevertheless, it appears reasonable to give the smallest possible IC volume (<100 mL) at a rate of <5 mL/sec after adequate intravascular hydration of the patient [3,4,7,9].

As allergists, it is essential to recognize this condition because these patients may be referred to our clinics due to the uniqueness of the case, limited available literature, and unfamiliarity with this pathology among various medical specialties. Occasionally, along with neurological symptoms, the appearance of cutaneous lesions [9] may occur, as in our case, where the patient presented with a late MPE. Therefore, an allergological study was suggested for our patient to identify the possible involvement of an immunological mechanism (hypersensitivity reaction) that could explain the associated skin symptoms.

The gold standard for a definitive allergological diagnosis would be a controlled challenge test with the suspected IC. However, in our case, the severity of the clinical presentation and the risk of recurrence upon re-exposure were sufficient reasons not to recommend it, as the potential risk of triggering a new episode of CIE did not justify the benefit of ruling out a possible allergy to IC. For this reason, the patient was advised to use alternative diagnostic and/or therapeutic methods, unless the benefit of using IC clearly outweighs the associated risks

Although the pathophysiology of CIE remains unclear, it seems reasonable to accept that the osmolarity of the IC, as well as its potential direct neurotoxic effect, are the most likely causes of this condition. However, in cases where symptoms associated with immediate or delayed hypersensitivity reactions arise—raising suspicion of an underlying immunological mechanism—a comprehensive allergological evaluation should be carried out in order to provide more appropriate guidance to the patient.

We present the case of a patient who developed CIE and an MPE a few hours after undergoing a thoracic angioCT with IC, which spontaneously and completely resolved after 48 hours, with normal neuroimaging studies and non-conclusive allergological evaluation. It is crucial to raise awareness of this condition among allergists for the proper management of these patients, as they may be referred to our practice.

## Patient consent

The patient was clearly informed that her privacy would be fully respected and that no personally identifiable information would be disclosed or published in any form. She understands that every effort will be made to ensure her anonymity.

Furthermore, the patient was given the opportunity to ask any questions regarding the publication of her case, including potential implications and dissemination through medical journals. All her concerns were addressed satisfactorily, and she has freely and voluntarily agreed to the publication.

## CRediT authorship contribution statement

Patricia Andrade Garbán and Marta Goyanes Malumbres performed the literature search and wrote the manuscript. Carolina Calvo Corbella, Raquel Izquierdo Fernández and Diego Mayo Canalejo looked after the patient, and performed the diagnostic workup. María González Labrador downloaded data from the clinical records system, and contributed to the literature review. Sabela Pérez Codesido contributed to the literature review and the diagnostic workup. Miguel Ángel Tejedor contributed to the literature review and performed the diagnostic workup. All authors undertook a critical review of the manucript.
